# Prognostic significance of clonal hematopoiesis in STEMI: a 10-year follow-up reveals high-risk gene mutations

**DOI:** 10.1186/s40246-025-00757-2

**Published:** 2025-05-12

**Authors:** Wen-Lang Fan, Jih-Kai Yeh, Li-Ching Hsieh, Ming-Lung Tsai, Ming-Yun Ho, Yi-Chun Huang, I-Chang Hsieh, Ming-Shien Wen, Chao-Yung Wang

**Affiliations:** 1 Department of Medical Research, Kaohsiung Chang Gung Memorial Hospital, Kaohsiung, Taiwan; 2 Division of Cardiology, Linkou Medical Center, Chang Gung Memorial Hospital, Taoyuan City, Taiwan; 3 School of Medicine, College of Medicine, Chang Gung University, Taoyuan City, Taiwan; 4 Graduate Institute of Genomics and Bioinformatics, National Chung Hsing University, Taichung, Taiwan; 5 Division of Cardiology, New Taipei Municipal TuCheng Hospital, New Taipei, Taiwan; 6 Institute of Cellular and System Medicine, National Health Research Institutes, Zhunan, Taiwan; 7 Department of Medical Science, National Tsing Hua University, Hsinchu, Taiwan

**Keywords:** Clonal hematopoiesis, ST-segment elevation myocardial infarction, Major adverse cardiovascular events

## Abstract

**Background:**

To elucidate the extent and clinical implications of clonal hematopoiesis of indeterminate potential (CHIP) prevalence in patients with ST-segment elevation myocardial infarction (STEMI), and to evaluate its utility as a contributory factor for risk stratification in long-term outcomes.

**Methods:**

Whole-exome sequencing was performed in a cohort of 101 patients presenting with STEMI who underwent emergency percutaneous coronary intervention. These patients were longitudinally followed for over 120 months. Their genomic data were compared with those from a control group of 706 individuals without cardiovascular events. Comparative analyses were conducted to identify patterns of CHIP between the STEMI and control cohorts.

**Results:**

In our cohort, 37.6% (n = 38) of STEMI patients exhibited somatic mutations associated with CHIP at a variant allele frequency of 1% or greater, compared to 22.8% (n = 161) in the control group. The most frequently detected mutations in STEMI patients were in the *ASXL1* and *CREBBP* genes, each present in 5.0% of this cohort. Long-term follow-up revealed that STEMI patients with CHIP had a higher incidence of major adverse cardiovascular events (MACEs), with an adjusted hazard ratio of 2.23 (95% confidence interval (CI) 1.16–4.28, p = 0.015).

**Conclusion:**

CHIP is prevalent in the STEMI patient cohort and is significantly correlated with adverse clinical outcomes. Incorporating CHIP status could enhance the risk stratification process, thus informing more tailored clinical management strategies for STEMI patients.

**Supplementary Information:**

The online version contains supplementary material available at 10.1186/s40246-025-00757-2.

## Introduction

Clonal hematopoiesis (CH) represents the expansion of blood cells harboring specific driver mutations in people without hematologic cancers [1, 2]. The most commonly mutated genes in CH are *DNMT3 A, TET2, ASXL1, and JAK2,* which confer a proliferative and survival advantage to mutated cells [3]. CH usually persists for a long period and rarely transforms into a clinical hematologic malignancy, with an incidence rate of 0.5–1% per year. CH is present in less than 1% of people under 35 years old but exists in 10–30% of healthy elderly individuals over 70 years old, a condition commonly termed clonal hematopoiesis of indeterminate potential (CHIP) [4]. Although CH is regarded as a pre-malignant condition, it is noteworthy that it is associated with an increased risk of cardiovascular diseases, including ischemic stroke and coronary heart events [5, 6].

Recent studies underscore the importance of CHIP in various cardiovascular scenarios, including cardiogenic shock during acute myocardial infarction (AMI) [7]. These investigations show that patients with CHIP mutations, specifically in *DNMT3 A or TET2* genes, face heightened risks, manifesting as worsened 30-day outcomes such as increased mortality or severe renal failure. Additionally, targeted amplicon sequencing in patients with severe aortic valve stenosis undergoing trans-femoral aortic valve implantation found that one-third had age-dependent CHIP-driver mutations, which, although not affecting other clinical metrics, were associated with a significant increase in medium-term all-cause mortality post-procedure [8]. Observational data also indicate that CHIP-related somatic mutations are commonly seen in patients with chronic heart failure, leading to unfavorable prognoses [9–11].

The linkage between these somatic mutations and cardiovascular disorders is further supported by studies in mice. In a *TET-2* mutated CH model, for example, monocyte-macrophages are activated with up-regulation of IL-1β signaling, mediated by the activated NLRP3 inflammasome, accelerating atherosclerosis development and maladaptive cardiac remodeling in mice [12–14].

ST-segment elevation myocardial infarction (STEMI) is mostly caused by atherosclerotic plaque rupture or erosion and subsequent thrombus formation, leading to acute total occlusion of an epicardial coronary vessel. Inflammation not only contributes to the vulnerable structural characteristics of plaque but also modulates the extent and severity of myocardial ischemic-reperfusion injury after the re-establishment of blood flow [15]. Widespread coronary inflammation observed in patients with acute coronary syndrome accounts for frequent recurrent thrombotic events during the subsequent period [16]. CHIP may play a critical role in promoting or remitting inflammatory reactions during and after acute myocardial infarction injury. Targeted deep sequencing of both *DNMT3 A* and *TET2* genes showed a 12.4% prevalence of these mutations among STEMI patients, correlating these with poor clinical outcomes, potentially mediated by elevated levels of inflammatory cytokines like IL-1β and IL-6 [17].

The targeted sequencing of *DNMT3 A* and *TET2* in current studies, while valuable, is limiting. This approach may obscure the contributions of other significant CHIP-related genes, such as *ASXL1* and *JAK2*. Adopting a more comprehensive technique like whole-exome sequencing could broaden our understanding of CHIP's role in cardiovascular events like STEMI. By capturing a wider array of mutations, we can uncover more intricate relationships between CHIP and adverse outcomes.

## Materials and methods

### Study design and patient enrollment

A prospective cohort study was conducted (NCT02674230), enrolling consecutive STEMI patients from a single 3500-bed tertiary university hospital starting July 9, 2011 [18]. Inclusion criteria were primary percutaneous coronary intervention (PCI) within 12 h of symptom onset and clear identification of the infarct-related artery. Exclusions included history of steroid use, prior hematological disorders, chronic kidney disease with GFR < 15 mL/min or on dialysis, BMI > 35 kg/m2, advanced liver cirrhosis (Child class C), and neuromuscular diseases. Blood samples were collected 28 days post-STEMI and, with patient consent, immediately post-PCI and at multiple time points thereafter. A control cohort was also established, featuring participants without prior major cardiovascular diseases. This control group comprised 706 individuals. The primary endpoint was major cardiovascular events (MACE), encompassing all-cause mortality, recurrent myocardial infarction, stroke, revascularization, and hospitalization for angina or heart failure. Approval was obtained from the Chang Gung Memorial Hospital Institutional Review Board, and informed consent was acquired from all participants.

### Sample preparation and whole-exome sequencing

DNA from STEMI patients and controls was sourced from peripheral blood mononuclear cells. DNA isolation was carried out using DNAeasy (Qiagen), adhering to the manufacturer's guidelines while avoiding vortexing to preserve DNA integrity. Genomic DNA will be extracted from peripheral venous blood samples. Exome capture was conducted using the Twist Comprehensive Exome panel (TWIST Technology, San Francisco, California, USA), and massively parallel sequencing was performed on the NovaSeq 6000 platform (Illumina, San Diego, CA) to yield paired-end 150-bp reads. Raw image analysis and base calling were executed with Illumina's default pipeline parameters. An average of 103 million reads was produced from STEMI patients, achieving an average and median coverage of 205.

### Mutation calling and variant filtering

Sequence data were aligned to the reference human genome (hg38) using the Burrows-Wheeler Aligner (BWA) [19], and duplicate reads were removed using Picard tools. We used the Genome Analysis ToolKit (GATK) [20] to perform the re-alignment and MuTect2 [20] to call somatic mutations (single nucleotide variants (SNV) and small insertions and deletions). Based on visual inspection using the Integrative Genomics Viewer (IGV), we established the following post-filtering criteria. First, we selected mutations covered by ≥ 20 reads in samples. We then processed different SNVs and indels. For SNVs, we selected mutations with a MuTect2 filter flag among “PASS”, “clustered_events” or “t_lod_fstar”. To improve specificity in the calling of mutations with low variant allele frequencies (VAF < 0.01) [21–23], we quantified the number of high-quality variant reads in the samples (mapping quality ≥ 50, base quality ≥ 20) and the number of variant reads in the sample. Only variants supported by ≥ 3 high-quality reads in samples were selected [24, 25]. For indels, we selected mutations with a MuTect2 filter flag among “PASS”, “clustered_events” or “str_contraction” supported by ≥ 2% reads in the sample.

### CHIP mutation screening

Using peripheral blood DNA with whole-exome sequencing, we searched for somatic mutations in 54 genes previously recognized as potential drivers of clonal hematopoiesis [5, 26–29]. CHIP was characterized by a clonal blood cell population carrying a CHIP-associated mutation with a variant allele frequency (VAF) ≥ 0.01. Our STEMI samples had an average coverage of 205X and a median of 185X (Figure S1). However, our CHIP mutation screening approach did not detect clonal skewing when mutations were absent in the suspected candidate driver genes.

### Manuscript preparation

In the preparation of this manuscript, the author(s) utilized Perplexity to facilitate efficiency in technical editing, linguistic refinement, and adherence to formatting standards. The AI-assisted technologies were employed solely to optimize clarity and precision in the presentation of the research, without influencing the intellectual content, methodology, or scientific conclusions. Following the use of these tools, the author(s) conducted a thorough review and made all necessary adjustments to ensure the manuscript's integrity, scholarly rigor, and alignment with the study's objectives. The author(s) take full responsibility for the accuracy, originality, and final content of the publication.

### Statistical analysis

Continuous variables were presented as median ± interquartile range (IQR) and were compared between groups with the Mann–Whitney U test. Categorical variables are presented as percentages and were compared using the Pearson chi-square test. To compare the MACE rates between patients with and without CHIP variants, proportional hazard assumptions were tested and adjusted in the multivariable analysis, which included the following variables: age, sex, comorbidity status, Killip classification on admission, discharge medications, laboratory levels of serum creatinine, low-density lipoprotein (LDL), peak troponin I, and echocardiographic assessment of left ventricular ejection fraction. We employed Kaplan–Meier survival analysis to estimate the survival functions according to CHIP carrier status and the specific CHIP gene group. Differences in survival distributions between the groups were assessed for statistical significance using the log-rank test. Moreover, the gene-specific effects on the study endpoint and dose–response association between the endpoint and the number of mutated genes were analyzed. Statistical significance was inferred at a P value < 0.05 for all analyses. All statistical analyses were performed in Stata version 17.0 (StataCorp).

## Results

### Prevalence and distribution of CHIP driver mutations

**Fig. 1:**
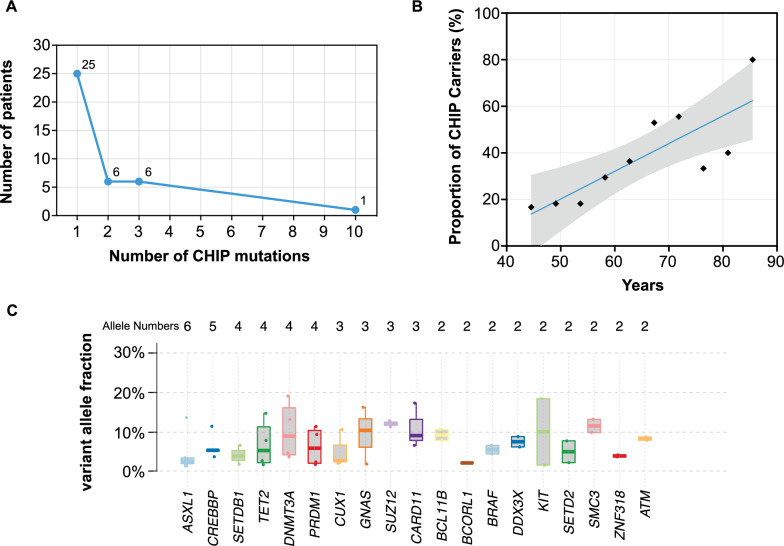
Distribution of CHIP mutations in relation to age in 101 STEMI patients. **A** Number of CHIP mutations per patient. **B** Frequency of CHIP increases with age. The data points represent the average proportion of CHIP carriers within each age bin. Age groups are binned in 5-year intervals, with each point corresponding to the mean proportion of CHIP carriers within that bin. The shaded area represents the 95% confidence interval around the fitted regression line, indicating the estimated range of the proportion of CHIP carriers within each age bin. **C** The distribution of CHIP-related mutations identified with VAF between 1 and 20% in the top 16 frequently mutated genes. The numbers of mutated allele are list at the top

In the study involving 101 STEMI patients and 706 control subjects eligible for whole- exome sequencing, 38 (37.6%) STEMI patients and 161 (22.8%) controls displayed at least one CHIP-associated mutation with a VAF of 1% or higher, based on the study's criteria for CHIP detection. Both groups included individuals with multiple CHIP mutations, ranging from 2 to 10 (Fig. 1A, Supplementary Table 1). Consistent with previous research, the prevalence of CHIP mutations increased with age (Fig. 1B, Figure S2). The most frequently affected CHIP genes among STEMI patients were *ASXL1*, *CREBBP*, *TET2*, *SETDB1*, and *DNMT3 A*, with percentages of 5.0% (n = 5), 5.0% (n = 5), 4.0% (n = 4), 4.0% (n = 4), and 3.0% (n = 3), respectively (Table 1). A comparison of gene frequencies between STEMI patients and controls revealed distinct CHIP mutation patterns, with *ASXL1* (0.85%, n = 6), *SUZ12* (0.14%, n = 1), and *PRDM1* (0.14%, n = 1) mutations being less common in controls than in STEMI patients (Table 1). Figure 1C provides a detailed summary of the mutations and their VAFs in both cohorts.

**Table 1:** The number of subjects with CHIP mutations in 101 STEMI patients and 706 controls

Gene	Number and frequency of patients with STEMI	Rank in patients	Number and frequency of control subjects	Rank in controls	Chi-square statistic with Yates correction	*P*-value
*ASXL1*	5 (4.95%)	1	6 (0.85%)	10	8.21	** < 0.01**
*CREBBP*	5 (4.95%)	1	21 (2.97%)	1	0.56	0.45
*TET2*	4 (3.96%)	3	14 (1.98%)	3	0.81	0.37
*SETDB1*	4 (3.96%)	3	20 (2.83%)	2	0.10	0.76
*DNMT3 A*	3 (2.97%)	5	13 (1.84%)	4	0.14	0.70
*SUZ12*	3 (2.97%)	5	1 (0.14%)	29	9.17	**< 0.01**
*PRDM1*	3 (2.97%)	5	1 (0.14%)	29	9.17	**< 0.01**
*CUX1*	3 (2.97%)	5	2 (0.28%)	24	6.46	0.01
*GNAS*	3 (2.97%)	5	7 (0.99%)	8	1.44	0.23
Total (51 genes)	38 (37.62%)		161 (22.80%)		9.22	**< 0.01**

STEMI ST segment elevated myocardial infarction

### Clinical characteristics of STEMI patients with and without CHIP detection

The average age of the STEMI cohort was 61 years, with females representing 10.9% of the group. Among these patients, 34.7% (n = 35) had dyslipidemia, 54.5% (n = 55) had hypertension, and 25.7% (n = 26) had diabetes mellitus. The mean left ventricular ejection fraction was 52.9%. Upon admission, 72.3% (n = 73) were classified as Killip class I, while 7.9% (n = 8) as class IV. The left descending artery was the infarct-related artery in more than half of the cohort. Most patients received standard guideline-recommended treatments: antiplatelets (94.1%), statins (83.2%), beta-blockers (86.1%), and angiotensin converting enzyme (ACE) inhibitors or angiotensin II receptor blockers (ARB) (74.3%), unless contraindicated. Table 2 presents baseline characteristics for patients with and without CHIP mutations. Those with CHIP mutations were marginally older (average ages of 62.7 vs 59.5 years); however, no significant differences were observed in demographic characteristics, cardiovascular risk factors, biochemical analyses, or clinical characteristics of STEMI between the two groups.

**Table 2:** Clinical characteristics of patients with STEMI according to the presence of CHIP mutations

Characteristics	Total	CHIP carriers	Non-carriers	*p*
Number (n)	101	37	64	
Age, y, mean ± SD [range]	61 ± 11.4 [38–89]	65.2 ± 10.9 [40–89]	58.6 ± 11.1 [38–89]	0.005
Gender (female), n (%)	11 (10.9%)	4(10.8%)	7 (10.9%)	> 0.999
Weight, kg, mean ± SD	71.7 ± 11.3	68.6 ± 10.8	73.3 ± 11.4	0.054
Height, cm, mean ± SD	163.8 ± 11.3	164.4 ± 6.8	163.5 ± 13.3	0.636
BMI, kg/m^2^, mean ± SD	27.6 ± 13.3	25.4 ± 3.4	28.9 ± 16.4	0.113
Dyslipidemia, n (%)	35 (34.7%)	9 (24.3%)	26 (40.6%)	0.129
Systemic hypertension, n (%)	55(54.5%)	21 (56.8%)	34 (53.1%)	0.836
Diabetes mellitus, n (%)	26 (25.7%)	8 (21.6%)	18 (28.1%)	0.637
Current smoker, n (%)	56 (55.4%)	21 (56.8%)	35 (54.7%)	> 0.999
SBP, mm Hg, mean ± SD	126.3 ± 25	123.2 ± 26.4	128 ± 24.1	0.358
Prior CABG, n (%)	0 (0%)	0 (0%)	0 (0%)	NA
Killip class on admissions, n (%)				0.436
I	73 (72.3%)	25 (67.6%)	48 (75%)	
II	10 (9.9%)	4 (10.8%)	6 (9.4%)	
III	10 (9.9%)	3 (8.1%)	7 (10.9%)	
IV	8 (7.9%)	5 (13.5%)	3 (4.7%)	
Infarct-related artery, n (%)				0.427
LAD	44 (43.6%)	12 (32.4%)	32 (50%)	
RCA	21 (20.8%)	8 (21.6%)	13 (20.3%)	
LCX	8 (7.9%)	3 (8.1%)	5 (7.8%)	
LM	1 (1%)	1 (2.7%)	0 (0%)	
LAD + RCA	12 (11.9%)	5 (13.5%)	7 (10.9%)	
RCA + LCX	7 (6.9%)	3 (8.1%)	4 (6.3%)	
LAD + LCX	8 (7.9%)	5 (13.5%)	3 (4.7%)	
Therapy after discharge, n (%)				
Aspirin	95 (94.1%)	44 (93.6%)	51 (96.2%)	> 0.999
Statin	84 (83.2%)	40 (85.1%)	44 (83%)	0.589
β-Blocker	87 (86.1%)	39 (83%)	48 (90.6%)	0.766
ACE inhibitors or ARB	75 (74.3%)	33 (70.2%)	42 (79.2%)	0.156
Biochemistry analysis at admission				
BUN, mg/dL, mean ± SD	19 ± 8.1	19.2 ± 9.2	18.8 ± 7.5	0.816
Creatinine, mg/dL, mean ± SD	1.1 ± 0.6	1.1 ± 0.4	1.1 ± 0.7	0.847
Total cholesterol, mg/dL, mean ± SD	168.6 ± 37.7	165.8 ± 35.9	170.3 ± 39.2	0.562
Triglyceride, mg/dL, mean ± SD	147 ± 75.9	152 ± 81	144.1 ± 73.3	0.627
LDL, mg/dL, mean ± SD	100.8 ± 34.5	100.5 ± 36.1	101 ± 33.8	0.941
HDL, mg/dL, mean ± SD	47.3 ± 39.4	45.1 ± 32	48.5 ± 43.4	0.647
Uric acid, mg/dL, mean ± SD	6.6 ± 1.7	6.8 ± 1.9	6.6 ± 1.6	0.48
Fasting glucose, mg/dL, mean ± SD	119.1 ± 45.2	120.3 ± 56.4	118.4 ± 38.2	0.857
Peak troponin I, ug/dL, mean ± SD	39.9 ± 186	28.2 ± 61.4	46.9 ± 231.4	0.552
LVEF, n (%)	52.9 ± 11.7	52 ± 12.6	53.4 ± 11.2	0.585

Values are presented as mean ± IQR

ACE, angiotensin-converting enzyme; ARB, angiotensin receptor blockers; BMI, body mass index; BUN, blood urea nitrogen CABG, coronary artery bypass graft; HDL, high density lipoprotein; IQR, interquartile range; LAD, left anterior descending coronary artery; LCX, left circumflex artery; LDL, low density lipoprotein; LM, left main; LVEF, left ventricular ejection fraction; RCA, right coronary artery; SBP, systolic blood pressure; STEMI, ST-segment elevated myocardial infarction

### Mutation types among CHIP associated genes

**Fig. 2:**
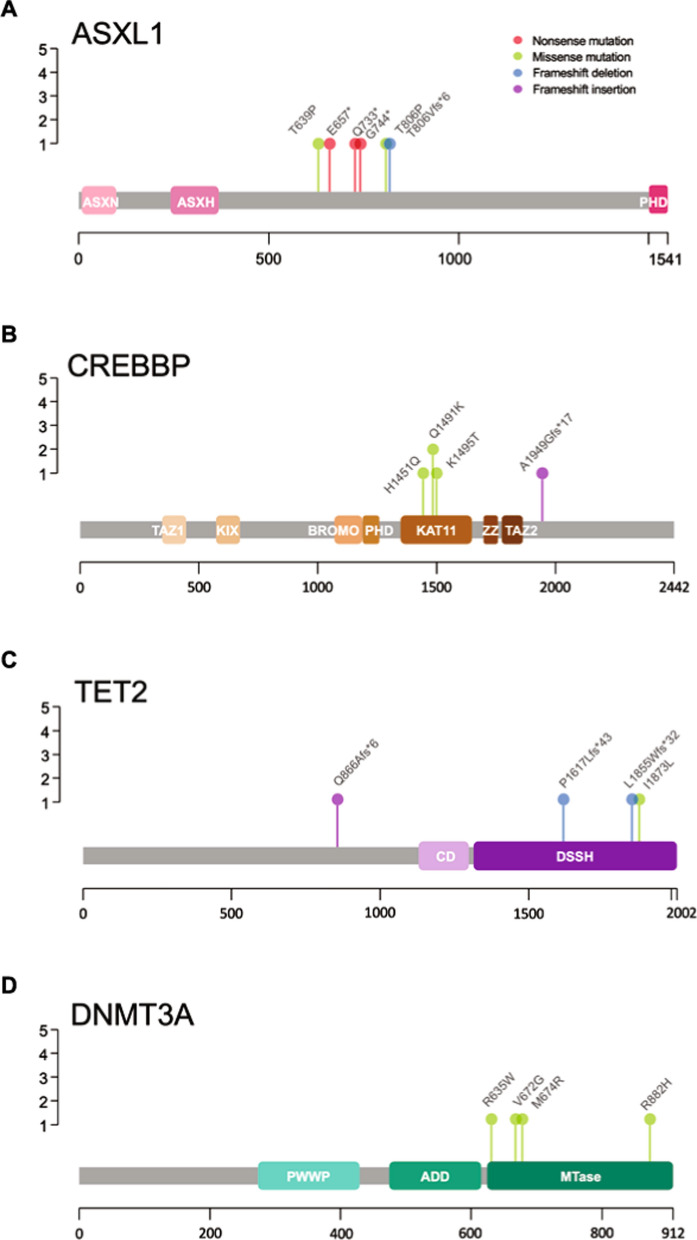
Predominant gene mutations identified in STEMI patients. **A–D** Lollipop plots for the proteins ASXL1, CREBBP, TET2, and DNMT3 A, showing CHIP mutations. Red lollipops represent nonsense mutations, while green lollipops signify missense mutations; violet indicate frameshift insertions; and blue indicate frameshift deletions. The height of each lollipop correlates with the frequency of mutations within the cohort

**Fig. 3:**
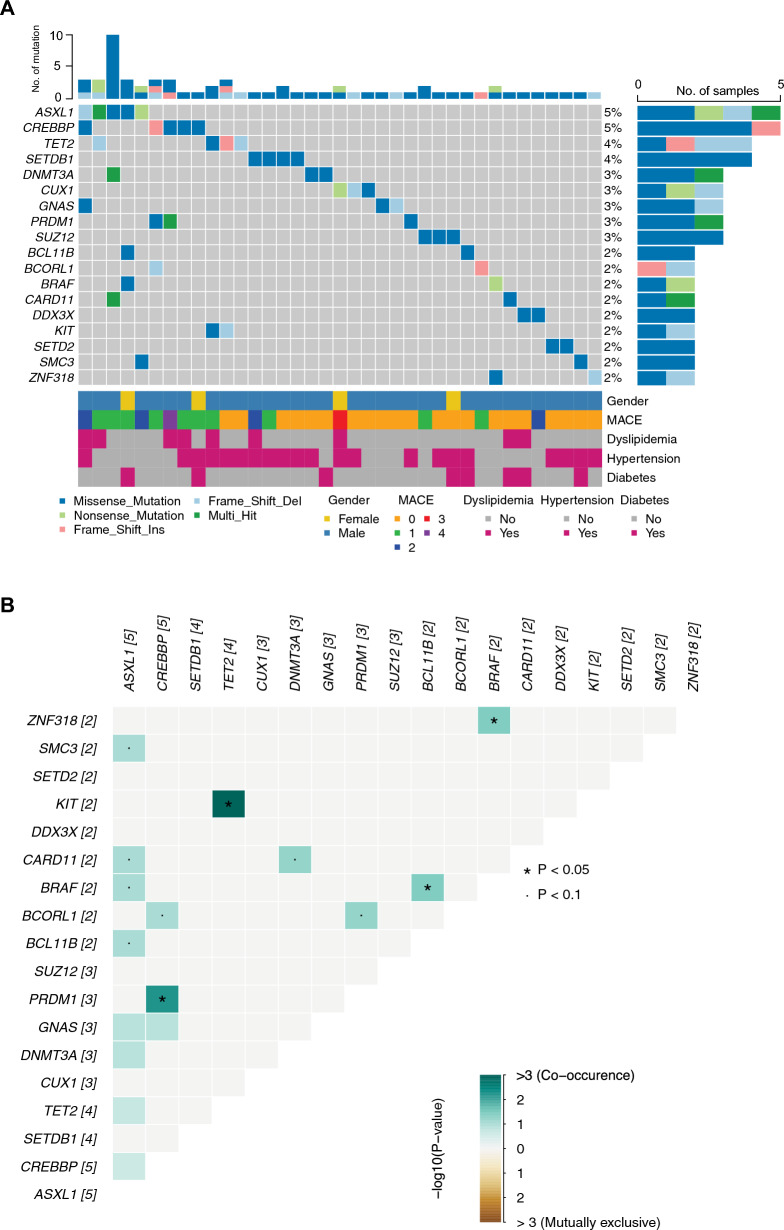
Genetic landscape of CHIP mutations in STEMI patients. **A** Oncoplot summarizes prevalent CHIP mutations among STEMI patients. Patients are represented in columns, and genes with specific mutations are represented in rows. Blue highlights CHIP mutations found in at least three patients (≥ 2.9% of the cohort). **B** Analysis of co-occurrence and exclusivity of genetic variants. Green indicates co-occurrence; brown indicates mutual exclusivity

The genetic variants selected as potential drivers of CHIP encompass SNV and frameshift insertions/deletions (indels), anticipated to cause significant functional impairments. Figure 2 delineates the mutation types and locations in CHIP's most frequently impacted genes within the STEMI cohort. Notably, *ASXL1* gene mutations comprise in-frame and frameshift deletions, along with nonsense and missense mutations. In contrast, the *CREBBP* gene predominantly exhibits missense mutations. Figure 2 and Supplementary Table 2 detail the somatic SNV and frameshift indels identified in CHIP-afflicted STEMI patients. Additionally, Fig. 3A presents an oncoplot that graphically represents the mutation distribution and frequency, emphasizing the mutation patterns and prevalence in *ASXL1* and *CREBBP* genes.

### Co-occurring genes with CHIP mutations in STEMI patients

In our examination of STEMI patients, we assessed the presence of concurrent and exclusive CHIP mutations. Using the'somaticInteractions'function in maftools, 101 whole-exome sequences were scrutinized, revealing significant genetic interactions. Specifically, a significant co-occurrence was identified between *TET2* and *KIT* mutations (Fig. 3B), as well as between *CREBBP* and *PRDM1* mutations. Our analysis found that 21.78% (22 out of 101) of the patients harbored concurrent mutations. In contrast, 30.69% (31 out of 101) exhibited exclusive mutations, indicating a substantial proportion of the cohort with unique genetic alterations impacting CHIP. No significant genetic interaction was found in the *ASXL1* gene.

### CHIP status and clinical outcomes

**Fig. 4:**
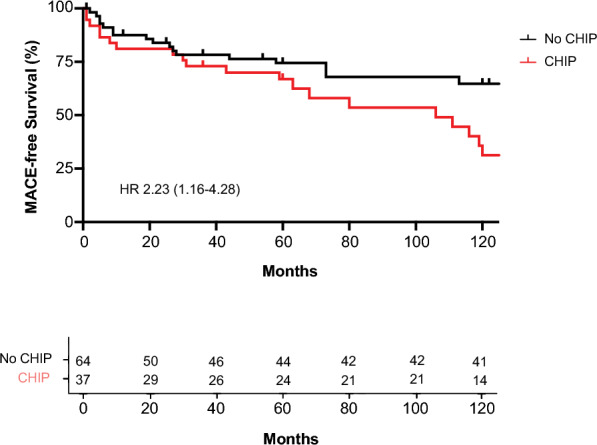
Impact of CHIP mutations on MACEs in STEMI patients. Kaplan–Meier curves showing MACE-free survival comparing patients with and without CHIP mutations. The HR with 95% confidence intervals is provided. Statistical significance was assessed by Log-rank (Mantel-Cox) test (p = 0.002)

**Fig. 5:**
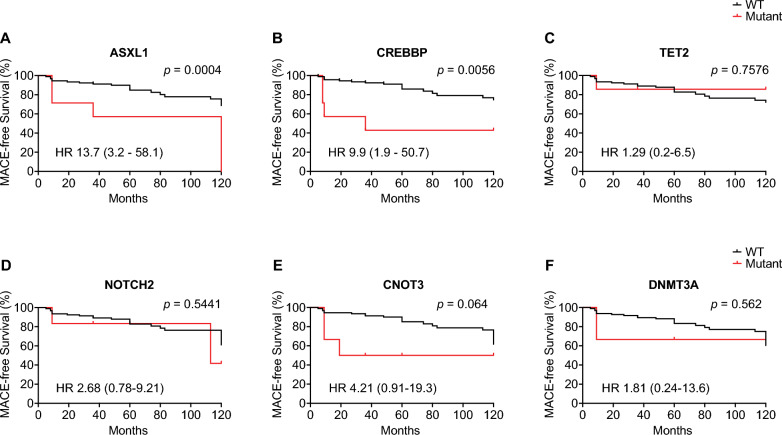
Clinical impact of specific somatic mutations on MACE-free survival in STEMI patients. Kaplan–Meier survival curves illustrate differences based on specific gene mutations associated with CHIP. HR and 95% confidence intervals are indicated. Log-rank tests assessed statistical significance

Over an average follow-up period of 120 months, 23 STEMI patients with CHIP and an equal number without CHIP reached the primary composite endpoint, which included death, ischemic stroke, and recurrent myocardial infarction. The presence of CHIP was associated with an increased risk of MACE, exhibiting a hazard ratio (HR) of 2.23 (95% confidence interval [CI] 1.16–4.28), following adjustments for age and sex (refer to Fig. 4). We conducted logistic Cox regression analyses to assess if particular genes impacted the prognostic relevance of CHIP, with Kaplan–Meier curves illustrating these outcomes (see Fig. 5). Of the CHIP-associated genes analyzed, only mutations in *ASXL1* and *CREBBP* showed a significant correlation with adverse clinical outcomes in STEMI patients, with *ASXL1* mutations associated with an HR of 13.7 (95% CI 3.2–58.1, p = 0.0004) and *CREBBP* mutations with an HR of 9.9 (95% CI 1.9–50.7, p = 0.0056).

## Discussion

This study on STEMI patients identified four principal findings. Firstly, the prevalent somatic mutations linked to CHIP in Asians differ from those in Caucasians, with the *CREBBP* and *ASXL1* genes being the most common in the Asian cohort. Secondly, CHIP was found in 37% of the STEMI patients, significantly correlating with higher risks of MACEs during prolonged follow-up. Thirdly, among the various CHIP mutations, *ASXL1* showed a higher prevalence in STEMI patients, suggesting some CHIP mutations may be more prevalent than others in this context. Lastly, the influence of specific CHIP mutations on clinical outcomes varied; notably, mutations in *ASXL1* and *CREBBP* were significantly associated with worse outcomes in STEMI patients. These findings reinforce CHIP's prognostic significance in cardiovascular diseases, indicating that diverse genetic mutations impact clinical outcomes through various pathophysiological mechanisms.

### Prevalence and prognostic impact of CHIP mutations

CH, in which mutated hematopoietic stem cells accumulate in the blood, increases in prevalence with age. Over 10% of healthy individuals over 70 years old carry these mutations [1, 4]. In contrast, CH is frequently observed in patients with advanced cardiovascular diseases. For instance, 18.5–38.7% of patients with ischemic or non-ischemic heart failure, 25.2–29% with cardiogenic shock, 12.4% with STEMI, and 33% undergoing transcatheter intervention for aortic valve stenosis have been reported to carry CHIP-associated mutations in various cohort studies. [7–11, 17].

Consistently, the presence of CHIP leads to poorer clinical outcomes, supporting its relevance in the pathophysiology of these cardiovascular diseases. In our STEMI cohort, the CHIP prevalence was 37.6%, correlated with a 2–threefold increased risk of MACEs. This frequency appears higher than in previous studies. Crucially, the prevalence of CH is shaped by the definition of mutations, the detection methods applied, and the choice of patient population. While CHIP mutations are traditionally identified with a VAF of at least 2%, existing research indicates that lower VAFs can also correlate with adverse outcomes in heart failure patients. In this research, we adopted a VAF threshold of ≥ 1% to inclusively capture CHIP mutations with functional impacts, leading to the identification of a higher prevalence rate. Moreover, the median sequencing depth for gene exons reached 205.4 reads, facilitating the accurate identification of rarer variants. Considering the dose–response relationship between clone size and clinical outcomes, reevaluating the optimal VAF threshold for clinical risk stratification across different cardiovascular conditions is essential.

Regarding to the prevalence of specific CHIP genes, Wang et al. [17] identified 12.4% of STEMI patients carrying mutations of *DNMT3 A* or *TET2* with a VAF > 2% and Böhme et al. [7] detected 29% of patients of advanced AMI with multivessel diseases and cardiogenic shock having mutations of *DNMT3 A*, *TET2*, *ASXL1*, and *JAK2* with a VAF > 2%.

We recognize that our finding of *ASXL1* and *CREBBP* as the most prevalently mutated genes in STEMI patients differs from previous reports, which often highlight *DNMT3 A* and *TET2* mutations as more common. There are several potential explanations for these differences. First, the prevalence of CHIP mutations can vary among different ethnic or regional populations. Common mutations detected in populations of European or North American descent are not necessarily the same as those in East Asian populations. The ethnic disparity may arise from a complex interplay of genetic, environmental, and socioeconomic factors. Environmental influences, including diet, pollution, and stress, can contribute to disease susceptibility through epigenetic modifications. Most CHIP-related genes, including *ASXL1*, *DNMT3 A*, and *CREBBP*, are known to play key roles in regulating epigenetic processes such as DNA methylation and histone acetylation. Consequently, different environmental exposures across populations may affect the incidence and progression of somatic mutations that contribute to clonal hematopoiesis [30]. Several studies have reported lower frequencies of *DNMT3 A* mutations in certain cohorts, supporting our findings. For example, Felicitas et al. [31] observed *DNMT3 A* mutations in only 2.6% of 193 myelodysplastic syndrome patients, while Hideki et al. [32] reported *ASXL1* as the most frequent mutation (30.8%) in a cohort of *SETBP1*-mutated myeloid malignancies, with *DNMT3 A* mutations found in only 11.5%. These variations suggest that CHIP mutation prevalence may differ across populations and disease settings. A practical illustration of the significance of ethnic differences is the East-Asian Paradox, where East Asians show higher platelet reactivity despite clopidogrel therapy but paradoxically lower rates of ischemic events than Caucasians. This is linked to a high prevalence of *CYP2 C19* loss-of-function alleles in East Asians [33], which reduces clopidogrel efficacy. Similarly, East Asians require lower doses of warfarin due to genetic variations in the *VKORC1* gene, while Caucasians and African Americans typically need higher doses [34]. Recognizing these disparities emphasizes the importance of integrating CHIP mutation profiles into population-specific disease prevention, risk assessment, and therapeutic strategies to optimize cardiovascular care outcomes globally.

Second, we employed whole-exome sequencing with a lower VAF threshold of ≥ 1% to detect CHIP mutations. This comprehensive approach allowed us to capture a broader range of mutations, including those with lower allele frequencies that might be missed by targeted sequencing methods or higher VAF thresholds used in other studies. In previous publications, increasing risks of clinical events, even short-term mortality, were observed in individuals carrying driver mutations with VAF 1–2%. Moreover, different driver mutations are thought to have varying magnitudes of clinical impacts. Mutations with less burden or smaller clones may not be"weak drivers"in disease initiation and progression. Besides, the use of whole-exome sequencing provides a more detailed and inclusive genetic landscape, which could explain the higher prevalence of *ASXL1* and *CREBBP* mutations in our cohort. We presumed it is possible that *ASXL1* and *CREBBP* mutations play a more prominent role in the pathophysiology of STEMI. Further research is needed to understand the functional impacts of these mutations. In fact, we observed that clonal hematopoiesis is common in patients with STEMI and has a clear impact on clinical outcomes. Comprehensive detection of target CHIP driver mutations can help us identify patients prone to serious consequences and intensify treatment accordingly in clinical practice.

### CHIP, inflammation, and thrombosis in pathophysiology of STEMI

CHIP is common in older individuals and is linked to increased cardiovascular mortality and atherosclerotic vascular diseases, although the mechanisms underlying this association remain unclear. Recent studies using genetically engineered mice have focused on how CHIP contributes to cardiovascular disease development. For instance, mice with *TET2* knockout bone marrow transplants showed accelerated atherosclerosis, activated through the NLRP-3/IL-1β inflammasome pathway and upstream IL-6 signaling [22]. Mice with *Jak2 V617 F* mutation developed extensive atheromas with neutrophil deposition, contributing to thrombosis [23]. The CANTOS study demonstrated fewer cardiovascular events with canakinumab treatment, particularly benefiting those with *TET2* CHIP [35]. Additionally, reduced IL-6 signaling mitigated cardiovascular risk in individuals with large CHIP clones (VAF > 10%) [25]. Heightened inflammatory responses in clones with mutated leukocytes are currently suggested as a predominant effect among those carrying CHIP mutations.

### Mechanisms of *CREBBP* and *ASXL1* in STEMI pathogenesis

CREBBP is a histone acetyltransferase that regulates gene expression by acetylating both histone and non-histone proteins. It functions as a transcriptional co-activator, influencing key biological processes, including hematopoiesis, cell differentiation, DNA repair, and immune surveillance. Through multiple mechanisms, CREBBP modulates immune responses by promoting regulatory T cell differentiation, macrophage polarization, and immune surveillance by natural killer cells. Mutations in *CREBBP* impair these processes, reducing the expression of major histocompatibility complex class II, diminishing antigen presentation, and facilitating immune evasion [36]. Additionally, *CREBBP* mutations activate pro-inflammatory pathways through epigenetic dysregulation, leading to exaggerated inflammation [37–39]. This has been linked to tissue fibrosis, including hepatic and myocardial fibrosis, by promoting fibroblast activation and extracellular matrix deposition [40].

ASXL1 is involved in epigenetic regulation through modifications such as histone H3 K27 me3 and H2 AK119Ub, which control genes related to cellular proliferation, differentiation, and apoptosis [41]. *ASXL1* mutations have been associated with a pro-thrombotic state, partly due to enhanced activation of the AIM2 inflammasome, resulting in elevated secretion of pro-inflammatory cytokines like IL-1β. Mutated *ASXL1* cells can promote vascular inflammation, altering immune responses and creating a thrombogenic environment [42]. Previous studies have reported an association between thrombotic events and mutations in DTA genes (*DNMT3 A*, *TET2*, and *ASXL1*), reinforcing their role in thrombogenesis [43].

In the context of STEMI, mutations in *CREBBP* and *ASXL1* may contribute to worse outcomes through complementary mechanisms. *CREBBP* mutations may drive plaque rupture by exacerbating chronic inflammation and promoting adverse post-infarction myocardial remodeling via fibrosis. *ASXL1* mutations may foster a pro-thrombotic state and enhance vascular inflammation, further increasing thrombotic risk and immune dysregulation. These dual mechanisms could explain the higher risk of adverse outcomes observed in STEMI patients with both *CREBBP* and *ASXL1* mutations in our cohort. The specific contributions of these mutated genes to the pathophysiological mechanisms underlying STEMI still need clarification. Current STEMI models consider a range of factors, including plaque characteristics, circulating biomarkers, and the response to myocardial injury [44]. Acute occlusion in STEMI can arise not only from vulnerable plaque rupture but also from a pro-thrombotic milieu [45]. The degree of reperfusion injury largely hinges on elements such as reactive oxygen species generation, inflammatory activity, and microvascular dysfunction [46, 47]. CH mutations remain to be explored whether they can prolong leukocyte lifespan, amplify inflammation, disrupt coagulation, and postpone immune cell polarization, which contributes to enlarged infarct sizes and adverse outcomes. Further studies exploring the role of CH in generating pro-inflammatory and pro-thrombogenic conditions in atherosclerotic vascular diseases are merited.

### Comparative analysis with existing cardiovascular genomics research

Our study expands on recent findings regarding CHIP as a risk factor for cardiovascular disease. Wang et al. identified CHIP mutations in 16.5% of STEMI patients, primarily focusing on *DNMT3 A* and *TET2* mutations with a VAF ≥ 2%, demonstrating increased risk of death or major adverse cardiac events [17]. In contrast, our study used whole-exome sequencing and a VAF threshold of ≥ 1%, allowing for the detection of a wider range of mutations, including *ASXL1* and *CREBBP*, and highlighting their significant impact on outcomes.

Dregoesc et al. also found that CHIP was linked to increased cardiovascular events in stable coronary artery disease patients, with a VAF cut-off of > 1.07%. They observed that CHIP mutations were associated with increased levels of tissue factor, PAR-1, and matrilysin, suggesting a role in thrombosis [48]. Our study builds on these findings by further demonstrating the significance of *ASXL1* and *CREBBP* mutations in acute settings like STEMI, underscoring that smaller CHIP clones (VAF ≥ 1%) may still be clinically relevant.

Both studies emphasize that other CHIP mutations beyond *DNMT3 A* and *TET2* may have prognostic value. Our study reveals that CHIP mutations, particularly *ASXL1* and *CREBBP*, are associated with worse outcomes in STEMI patients. These mutations likely promote plaque rupture, thrombus formation, and adverse post-infarction remodeling through mechanisms involving chronic inflammation and heightened thrombosis. This suggests that genetic testing for CHIP could play a significant role in improving cardiovascular risk stratification. Incorporating CHIP testing into clinical practice would allow early identification of high-risk patients, enabling clinicians to refine risk assessment beyond traditional factors. CHIP-positive patients with inflammatory and pro-thrombotic mutations might require more targeted interventions to reduce the likelihood of recurrent events. By detecting these mutations, we could better predict which patients are prone to adverse outcomes and tailor their treatment accordingly.

For CHIP-positive patients, modifying treatment strategies to address both inflammation and thrombosis is essential. Anti-inflammatory agents, such as those targeting IL-1β (e.g., canakinumab), could help reduce inflammatory responses that contribute to plaque instability and myocardial damage. Additionally, intensified anti-thrombotic regimens, such as dual antiplatelet therapy with ticagrelor or prasugrel, may provide better protection against recurrent ischemic events by overcoming potential resistance to medications like clopidogrel. These approaches could help mitigate the increased risks associated with CHIP mutations and improve both short-term and long-term outcomes in this patient population.

### Study limitations

Our study employed whole-exome sequencing, which enabled us to detect a broader range of CHIP mutations compared to previous studies that utilized targeted sequencing approaches. This comprehensive methodology allowed us to identify mutations with lower allele frequencies, providing a more detailed genetic landscape of STEMI patients. This is a significant strength, as it offers a deeper understanding of the genetic factors potentially contributing to adverse cardiovascular outcomes.

However, several limitations should be acknowledged. This is a prospective cohort study, and the procedure of primary percutaneous intervention as well as the prescription of medical therapy were at the discretion of the treating physician, which could introduce potential biases in the analysis of the relationship between CHIP status and clinical outcomes. Despite the sizable cohort, the sample size may still be insufficient to yield definitive conclusions given the heterogeneity among various CHIP driver mutations. Furthermore, our study was limited to an Asian population, which could affect the generalizability of our findings to other ethnic groups, particularly populations of European or North American descent. Additionally, the relatively low number of female participants may limit the applicability of the results across genders.

Another limitation is the absence of data on inflammatory biomarker levels, such as IL-6 and IL-1, in patients with and without CHIP presence. The variability of these biomarkers, influenced by factors such as timing of blood sampling, clinical presentation, interventional procedures, and acute-phase treatments, made consistent assessment challenging. Future studies should consider these biomarkers to better understand the inflammatory mechanisms underlying CHIP-associated cardiovascular risks. While our findings offer valuable insights, further large-scale, multi-ethnic studies are needed to validate our results and determine the broader applicability of CHIP as a biomarker for cardiovascular risk stratification.

## Conclusion

Our data illuminate the substantial prevalence of CHIP mutations, particularly in *ASXL1* and *CREBBP* genes, among STEMI patients. Notably, *ASXL1* mutations occur more frequently in STEMI cohorts compared to controls, underscoring its potential role in STEMI pathogenesis. These mutations significantly influence MACE-free survival, emphasizing the need for targeted risk stratification. However, the limited sample size calls for further, more extensive studies to corroborate our findings.

## Supplementary Information

Additional file 1: Figure S1. Sequencing depth of whole-exome sequencing analysis. The average and median sequencing depths of exons were 205.4 and 185.2, respectively

Additional file 2: Figure S2. Age distribution comparison between STEMI patients with and without CHIP mutations. The stacked bar graph illustrates the age distribution of STEMI patients stratified by CHIP mutation status. Patients with CHIP mutationswere significantly older than those without CHIP mutations. SD, standard deviation. CHIP, clonal hematopoiesis of indeterminate potential; MACE, major adverse cardiac events; HR, hazard ratio; STEMI, ST-segment elevation myocardial infarction.

Additional file 3: Supplementary Table 1. The number and distribution of CHIP mutations in 706 controls and 101 STEMI patients.

Additional file 4: Supplementary Table 2. Panel of 51 genes used to identify somatic driver mutations in clonal hematopoiesis.

## Data Availability

The clonal hematopoiesis whole-exome sequencing (CHIP-WES) datasets generated and/or analysed during the current study are not publicly deposited due to participant privacy constraints, but are available from the corresponding author on reasonable request.
