# Supplementary figures and images for: Prognostic significance of clonal hematopoiesis in STEMI: a 10-year follow-up reveals high-risk gene mutations
Source: Hum Genomics. 2025 May 12;19:51. doi: 10.1186/s40246-025-00757-2 (PMC12067743; doi:10.1186/s40246-025-00757-2)

Supplementary Figure 1

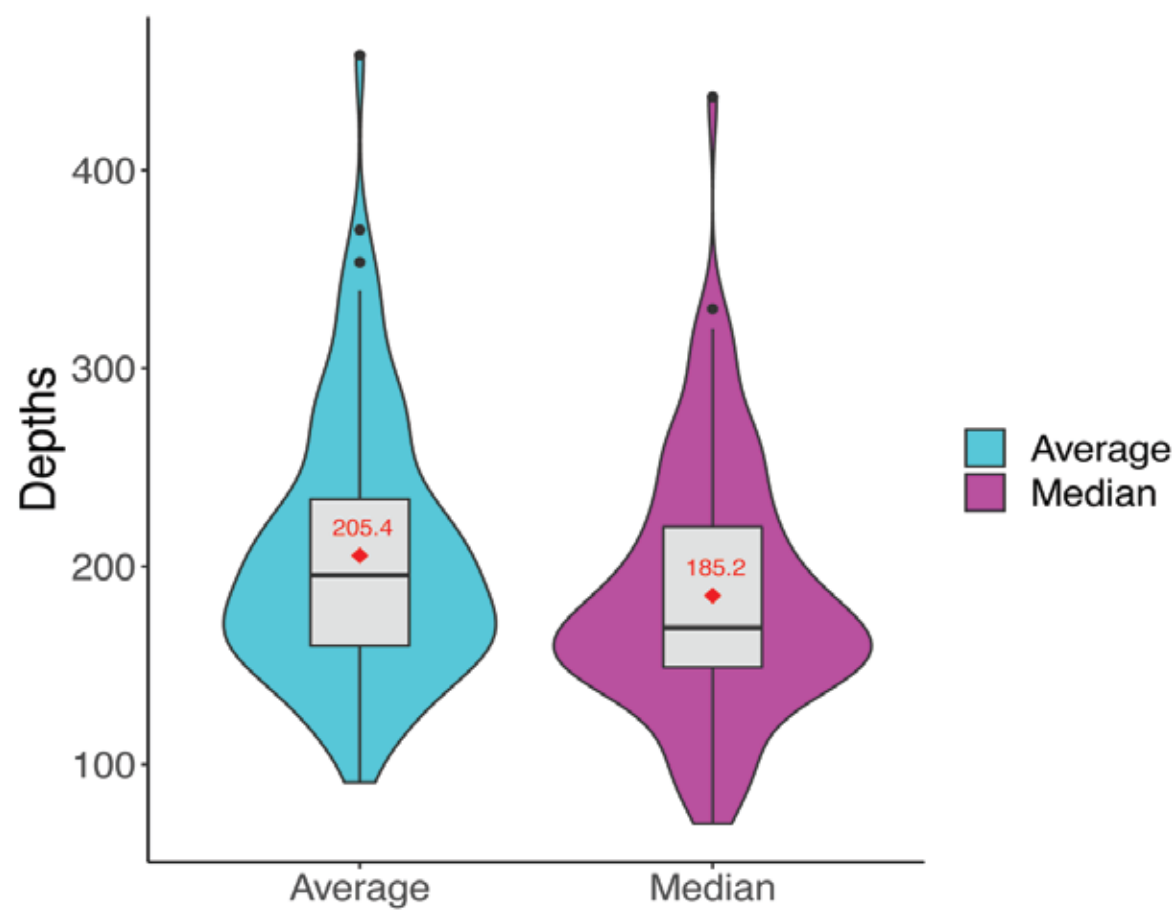

Supplement: Supplementary file 1 — Additional file 1: Figure S1. Sequencing depth of whole-exome sequencing analysis. The average and median sequencing depths of exons were 205.4 and 185.2, respectively [file 40246_2025_757_MOESM1_ESM.pdf]

## Stacked Age Distribution

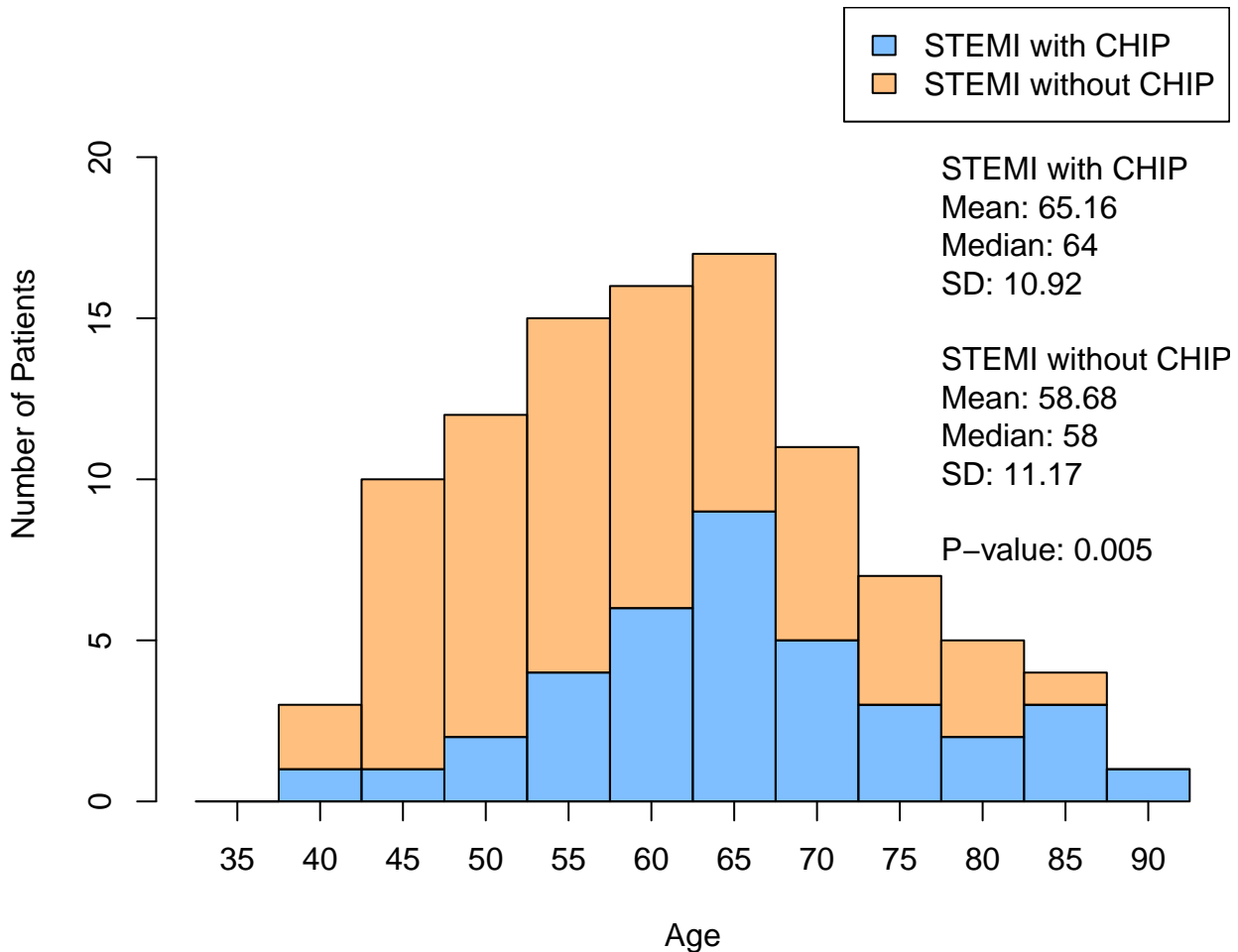

Supplement: Supplementary file 2 — Additional file 2: Figure S2. Age distribution comparison between STEMI patients with and without CHIP mutations. The stacked bar graph illustrates the age distribution of STEMI patients stratified by CHIP mutation status. Patients with CHIP mutationswere significantly older than those without CHIP mutations. SD, standard deviation. CHIP, clonal hematopoiesis of indeterminate potential; MACE, major adverse cardiac events; HR, hazard ratio; STEMI, ST-segment elevation myocardial infarction. [file 40246_2025_757_MOESM2_ESM.pdf]
