# Supplementary material for: Prognostic significance of clonal hematopoiesis in STEMI: a 10-year follow-up reveals high-risk gene mutations
Source: Hum Genomics. 2025 May 12;19:51. doi: 10.1186/s40246-025-00757-2 (PMC12067743; doi:10.1186/s40246-025-00757-2)
Supplement: Supplementary file 3 — Additional file 3: Supplementary Table 1. The number and distribution of CHIP mutations in 706 controls and 101 STEMI patients. [file 40246_2025_757_MOESM3_ESM.docx]

**Supplementary table 1.** The number and distribution of CHIP mutations in 706 controls and 101 STMEIs.

| Number of CHIP | Number and frequency of samples in 101 STEMI | Number and frequency of samples in 706 controls |
| --- | --- | --- |
| 1 | 25 (24.75%) | 132 (18.70%) |
| 2 | 6 (5.94%) | 22 (3.12%) |
| 3 | 6 (5.94%) | 3 (0.42%) |
| 4 | 0 (0%) | 3 (0.42%) |
| 5 | 0 (0%) | 0 (0%) |
| 6 | 0 (0%) | 0 (0%) |
| 7 | 0 (0%) | 0 (0%) |
| 8 | 0 (0%) | 1 (0.14%) |
| 9 | 0 (0%) | 0 (0%) |
| 10 | 1 (0.99%) | 0 (0%) |
| Total | 38 (37.62%) | 161 (22.80%) |
