# Supplementary material for: Prognostic significance of clonal hematopoiesis in STEMI: a 10-year follow-up reveals high-risk gene mutations
Source: Hum Genomics. 2025 May 12;19:51. doi: 10.1186/s40246-025-00757-2 (PMC12067743; doi:10.1186/s40246-025-00757-2)
Supplement: Supplementary file 4 — Additional file 4: Supplementary Table 2. Panel of 51 genes used to identify somatic driver mutations in clonal hematopoiesis. [file 40246_2025_757_MOESM4_ESM.docx]

**Supplementary table 2.**

A list of 51 genes utilized to identify somatic mutations driving clonal hematopoiesis.

| *ASXL1* | *FLT3* | *PTPN11* |
| --- | --- | --- |
| *ATM* | *GNAS* | *RAD21* |
| *BCL11B* | *GNB1* | *SETD2* |
| *BCOR* | *IDH2* | *SETDB1* |
| *BCORL1* | *IKZF1* | *SF1* |
| *BIRC3* | *JAK2* | *SF3B1* |
| *BRAF* | *KDM6A* | *SMC3* |
| *BRCC3* | *KIT* | *SRSF2* |
| *CARD11* | *KRAS* | *STAT3* |
| *CBL* | *LUC7L2* | *SUZ12* |
| *CD58* | *KMT2D*( *MLL2*) | *TET2* |
| *CD79B* | *MPL* | *TNFAIP3* |
| *CREBBP* | *MYD88* | *TNFRSF14* |
| *CUX1* | *NRAS* | *TP53* |
| *DDX3X* | *PPM1D* | *U2AF1* |
| *DNMT3A* | *PRDM1* | *ZNF318* |
| *EZH2* | *PRPF40B* | *ZRSR2* |

**List of genetic variants identified as drivers of clonal hematopoiesis in the STEMI patient cohort.**

| Patient ID | Hugo Symbol | Chr. | Position | Variant Type | Variant Classification | Ref. Allele | Alt. Allele | Ref. Count | Alt. Count | VAF | Ensembl Transcript ID | DNA Change | Protein Change |
| --- | --- | --- | --- | --- | --- | --- | --- | --- | --- | --- | --- | --- | --- |
| ami007 | ZNF318 | 6 | 43340445 | substitution | Missense_Mutation | CA | TG | 161 | 6 | 3.6% | ENST00000361428 | c.3552_3553inv | p.Gly1185Ser |
| ami007 | BRAF | 7 | 140734755 | SNV | Nonsense_Mutation | C | A | 88 | 4 | 4.3% | ENST00000496384 | c.2143G>T | p.Glu715Ter |
| ami026 | DDX3X | X | 41341595 | SNV | Missense_Mutation | G | A | 52 | 5 | 8.8% | ENST00000644876 | c.263G>A | p.Arg88His |
| ami027 | SETDB1 | 1 | 150950963 | substitution | Missense_Mutation | GAG | CTC | 400 | 7 | 1.7% | ENST00000271640 | c.2086_2088inv | p.Glu696Leu |
| ami039 | KIT | 4 | 54728031 | SNV | Missense_Mutation | C | T | 133 | 30 | 18.4% | ENST00000288135 | c.1900C>T | p.Arg634Trp |
| ami039 | TET2 | 4 | 105276127 | SNV | Missense_Mutation | A | C | 284 | 24 | 7.8% | ENST00000540549 | c.5617A>C | p.Ile1873Leu |
| ami041 | PRDM1 | 6 | 106105808 | SNV | Missense_Mutation | A | C | 172 | 22 | 11.3% | ENST00000369096 | c.1648A>C | p.Ile550Leu |
| ami052 | DNMT3A | 2 | 25234373 | SNV | Missense_Mutation | C | T | 214 | 8 | 3.6% | ENST00000264709 | c.2645G>A | p.Arg882His |
| ami055 | DNMT3A | 2 | 25243931 | SNV | Missense_Mutation | G | A | 165 | 8 | 4.6% | ENST00000264709 | c.1903C>T | p.Arg635Trp |
| ami069 | SETDB1 | 1 | 150950962 | SNV | Missense_Mutation | T | G | 315 | 22 | 6.5% | ENST00000271640 | c.2085T>G | p.Asn695Lys |
| ami082 | BRAF | 7 | 140924645 | SNV | Missense_Mutation | T | G | 200 | 14 | 6.5% | ENST00000496384 | c.59A>C | p.Asn20Thr |
| ami082 | BCL11B | 14 | 99271167 | SNV | Missense_Mutation | T | G | 380 | 45 | 10.6% | ENST00000357195 | c.52A>C | p.Ile18Leu |
| ami082 | ASXL1 | 20 | 32434627 | SNV | Missense_Mutation | A | C | 418 | 8 | 1.9% | ENST00000375687 | c.1915A>C | p.Thr639Pro |
| ami109 | CARD11 | 7 | 2928662 | SNV | Missense_Mutation | T | G | 386 | 27 | 6.5% | ENST00000396946 | c.1690A>C | p.Thr564Pro |
| ami117 | PRDM1 | 6 | 106105808 | SNV | Missense_Mutation | A | C | 195 | 20 | 9.3% | ENST00000369096 | c.1648A>C | p.Ile550Leu |
| ami117 | CREBBP | 16 | 3729203 | insertion | Frame_Shift_Ins | - | G | 106 | 6 | 5.4% | ENST00000262367 | c.5843dup | p.Ala1949GlyfsTer17 |
| ami117 | BCORL1 | X | 130037429 | deletion | Frame_Shift_Del | CTACACA | - | 141 | 3 | 2.1% | ENST00000540052 | c.4368_4374del | p.Tyr1457ProfsTer14 |
| ami131 | GNAS | 20 | 58854505 | SNV | Missense_Mutation | G | T | 26 | 3 | 10.3% | ENST00000371100 | c.1240G>T | p.Gly414Trp |
| ami133 | PRDM1 | 6 | 106105525 | SNV | Missense_Mutation | C | G | 414 | 7 | 1.7% | ENST00000369096 | c.1365C>G | p.Ser455Arg |
| ami133 | PRDM1 | 6 | 106105528 | insertion | Frame_Shift_Ins | - | C | 382 | 9 | 2.3% | ENST00000369096 | c.1372dup | p.His458ProfsTer54 |
| ami133 | CREBBP | 16 | 3736739 | SNV | Missense_Mutation | G | T | 349 | 19 | 5.2% | ENST00000262367 | c.4471C>A | p.Gln1491Lys |
| ami141 | CUX1 | 7 | 102197056 | SNV | Nonsense_Mutation | G | T | 182 | 5 | 2.7% | ENST00000360264 | c.1678G>T | p.Glu560Ter |
| ami141 | FLT3 | 13 | 28018523 | SNV | Missense_Mutation | C | A | 107 | 4 | 3.6% | ENST00000241453 | c.2485G>T | p.Asp829Tyr |
| ami142 | CREBBP | 16 | 3738600 | SNV | Missense_Mutation | A | C | 148 | 19 | 11.4% | ENST00000262367 | c.4353T>G | p.His1451Gln |
| ami152 | CUX1 | 7 | 102239510 | deletion | Frame_Shift_Del | A | - | 298 | 6 | 2.0% | ENST00000360264 | c.3850del | p.Thr1284ProfsTer11 |
| ami183 | CUX1 | 7 | 102277967 | SNV | Missense_Mutation | C | G | 34 | 4 | 10.5% | ENST00000437600 | c.1576C>G | p.His526Asp |
| ami192 | SF1 | 11 | 64767660 | SNV | Missense_Mutation | T | G | 374 | 11 | 2.9% | ENST00000377387 | c.1628A>C | p.Asn543Thr |
| ami192 | SUZ12 | 17 | 31947682 | SNV | Missense_Mutation | A | C | 175 | 24 | 12.1% | ENST00000322652 | c.452A>C | p.His151Pro |
| ami207 | ZNF318 | 6 | 43342120 | deletion | Frame_Shift_Del | G | - | 141 | 6 | 4.1% | ENST00000361428 | c.3368del | p.Pro1123LeufsTer33 |
| ami212 | DDX3X | X | 41357989 | substitution | Missense_Mutation | AG | CT | 46 | 3 | 6.1% | ENST00000625837 | c.2122_2123inv | p.Arg708Leu |
| ami218 | SMC3 | 10 | 110596523 | SNV | Missense_Mutation | A | C | 129 | 14 | 9.8% | ENST00000361804 | c.2089A>C | p.Asn697His |
| ami218 | ASXL1 | 20 | 32434681 | SNV | Nonsense_Mutation | G | T | 285 | 6 | 2.1% | ENST00000375687 | c.1969G>T | p.Glu657Ter |
| ami219 | SUZ12 | 17 | 31947682 | SNV | Missense_Mutation | A | C | 118 | 15 | 11.3% | ENST00000322652 | c.452A>C | p.His151Pro |
| ami221 | CREBBP | 16 | 3736739 | SNV | Missense_Mutation | G | T | 340 | 19 | 5.3% | ENST00000262367 | c.4471C>A | p.Gln1491Lys |
| ami253 | SUZ12 | 17 | 31947682 | SNV | Missense_Mutation | A | C | 102 | 15 | 12.8% | ENST00000322652 | c.452A>C | p.His151Pro |
| ami261 | BCORL1 | X | 130025047 | insertion | Frame_Shift_Ins | - | CTTCTGTGGGGAAGACTTCCTGG | 146 | 3 | 2.0% | ENST00000540052 | c.3750_3751insTGTGGGGAAGACTTCCTGGCTTC | p.Pro1251CysfsTer16 |
| ami264 | GNAS | 20 | 58853387 | deletion | Frame_Shift_Del | CT | - | 278 | 5 | 1.8% | ENST00000371100 | c.122_123del | p.Ala41GlyfsTer3 |
| ami277 | TET2 | 4 | 105275360 | deletion | Frame_Shift_Del | CT | - | 245 | 4 | 1.6% | ENST00000540549 | c.4850_4851del | p.Pro1617LeufsTer43 |
| ami277 | ASXL1 | 20 | 32434909 | SNV | Nonsense_Mutation | C | T | 319 | 11 | 3.3% | ENST00000375687 | c.2197C>T | p.Gln733Ter |
| ami277 | ASXL1 | 20 | 32434942 | SNV | Nonsense_Mutation | G | T | 340 | 10 | 2.9% | ENST00000375687 | c.2230G>T | p.Gly744Ter |
| ami288 | NRAS | 1 | 114709602 | substitution | Missense_Mutation | AA | CC | 175 | 11 | 5.9% | ENST00000369535 | c.416_417delinsGG | p.Ile139Arg |
| ami288 | DNMT3A | 2 | 25241623 | SNV | Missense_Mutation | A | C | 232 | 35 | 13.1% | ENST00000264709 | c.2021T>G | p.Met674Arg |
| ami288 | DNMT3A | 2 | 25241629 | SNV | Missense_Mutation | A | C | 212 | 50 | 19.1% | ENST00000264709 | c.2015T>G | p.Val672Gly |
| ami288 | CARD11 | 7 | 2944355 | SNV | Missense_Mutation | A | C | 201 | 42 | 17.3% | ENST00000396946 | c.541T>G | p.Tyr181Asp |
| ami288 | CARD11 | 7 | 2944358 | SNV | Missense_Mutation | A | C | 222 | 22 | 9.0% | ENST00000396946 | c.538T>G | p.Tyr180Asp |
| ami288 | LUC7L2 | 7 | 139409606 | SNV | Missense_Mutation | T | G | 90 | 15 | 14.3% | ENST00000354926 | c.731T>G | p.Leu244Arg |
| ami288 | ATM | 11 | 108256242 | SNV | Missense_Mutation | T | G | 94 | 8 | 7.8% | ENST00000278616 | c.2152T>G | p.Cys718Gly |
| ami288 | ATM | 11 | 108256244 | substitution | Missense_Mutation | TT | GG | 95 | 9 | 8.7% | ENST00000278616 | c.2154_2155delinsGG | p.Cys718_Ser719delinsTrpAla |
| ami288 | IDH2 | 15 | 90090590 | substitution | Missense_Mutation | TT | GG | 200 | 31 | 13.4% | ENST00000330062 | c.261_262delinsCC | p.Asn88His |
| ami288 | ASXL1 | 20 | 32435128 | SNV | Missense_Mutation | A | C | 191 | 30 | 13.6% | ENST00000375687 | c.2416A>C | p.Thr806Pro |
| ami292 | CREBBP | 16 | 3736725 | substitution | Missense_Mutation | CT | GG | 213 | 8 | 3.6% | ENST00000262367 | c.4484_4485delinsCC | p.Lys1495Thr |
| ami292 | ASXL1 | 20 | 32435127 | deletion | Frame_Shift_Del | CACCGTTCCTGCAGACAAT | - | 310 | 4 | 1.3% | ENST00000375687 | c.2415_2433del | p.Thr806ValfsTer6 |
| ami292 | GNAS | 20 | 58855270 | SNV | Missense_Mutation | A | C | 212 | 41 | 16.2% | ENST00000371100 | c.2005A>C | p.Ile669Leu |
| ami302 | KIT | 4 | 54695553 | deletion | Frame_Shift_Del | CCAT | - | 259 | 4 | 1.5% | ENST00000288135 | c.120_123del | p.His40GlnfsTer6 |
| ami302 | TET2 | 4 | 105236536 | insertion | Frame_Shift_Ins | - | G | 250 | 43 | 14.7% | ENST00000540549 | c.2595dup | p.Gln866AlafsTer6 |
| ami302 | PRPF40B | 12 | 49635954 | SNV | Missense_Mutation | A | C | 187 | 18 | 8.8% | ENST00000548825 | c.1387A>C | p.Met463Leu |
| ami305 | SETDB1 | 1 | 150950962 | SNV | Missense_Mutation | T | G | 291 | 12 | 4.0% | ENST00000271640 | c.2085T>G | p.Asn695Lys |
| ami305 | SF3B1 | 2 | 197423917 | SNV | Missense_Mutation | T | G | 223 | 45 | 16.8% | ENST00000335508 | c.86A>C | p.Gln29Pro |
| ami318 | SETD2 | 3 | 47057042 | SNV | Missense_Mutation | C | G | 230 | 5 | 2.1% | ENST00000409792 | c.6742G>C | p.Val2248Leu |
| ami340 | SMC3 | 10 | 110596523 | SNV | Missense_Mutation | A | C | 99 | 15 | 13.2% | ENST00000361804 | c.2089A>C | p.Asn697His |
| ami353 | SETDB1 | 1 | 150960905 | SNV | Missense_Mutation | A | C | 184 | 7 | 3.7% | ENST00000271640 | c.2843A>C | p.His948Pro |
| ami354 | TNFRSF14 | 1 | 2561708 | SNV | Missense_Mutation | C | G | 270 | 3 | 1.1% | ENST00000355716 | c.587C>G | p.Thr196Ser |
| ami357 | SETD2 | 3 | 47057347 | SNV | Missense_Mutation | A | C | 373 | 31 | 7.7% | ENST00000409792 | c.6437T>G | p.Met2146Arg |
| ami386 | BCL11B | 14 | 99175509 | SNV | Missense_Mutation | T | G | 332 | 28 | 7.8% | ENST00000357195 | c.1327A>C | p.Ile443Leu |
| ami392 | TET2 | 4 | 105276070 | deletion | Frame_Shift_Del | T | - | 554 | 15 | 2.6% | ENST00000540549 | c.5562del | p.Leu1855TrpfsTer32 |

Chr.=Chromosome; Ref.=reference; Alt.=Alternate; SNV= single nucleotide variant; VAF= variant allele fraction.
